# The Influence of Chalazion History on Meibomian Gland Loss and DED

**DOI:** 10.1155/joph/9418834

**Published:** 2026-07-25

**Authors:** Ying Liu, Ying Zhou, Chong Xu, Tu Su

**Affiliations:** ^1^ Department of Ophthalmology, Shanghai Tenth People’s Hospital, School of Medicine, Tongji University, Shanghai 200072, China, tongji.edu.cn; ^2^ Department of Ophthalmology, Xinjiang 474 Hospital, Urumqi 830011, Xinjiang, China; ^3^ Department of Ophthalmology, Shenzhen Nanshan District Maternity & Child Healthcare Hospital, Shenzhen 518052, Guangdong, China

**Keywords:** chalazion, comprehensive ocular surface analysis, dry eye disease, meibomian gland loss

## Abstract

**Objective:**

To investigate the impact of chalazion history on meibomian gland loss and dry eye disease (DED).

**Methods:**

This study was a retrospective case–control study. Sixty patients with a history of chalazion admitted to a certain hospital from June 2021 to June 2024, who were selected as the previous chalazion group, and another 90 patients with no previous history of chalazion who visited the outpatient clinic consecutively during the same period were selected as the nonchalazion group. Comprehensive ocular surface parameters were compared between groups to evaluate long‐term meibomian gland loss and DED. The chalazion group was further divided into MG loss (*n* = 42) and non‐MG loss (*n* = 18) subgroups based on glandular loss status. Logistic regression analyzed factors influencing meibomian gland loss. The MG loss subgroup was stratified by severity into mild–moderate (< 1/3 or 1/3–2/3 loss) and severe (> 2/3 loss) groups for comparison of parameters of DED (Schirmer’s test, tear break‐up time [BUT], and fluorescein staining [FL]). Pearson’s correlation assessed the relationship between meibomian gland loss severity and DED manifestations.

**Results:**

Significant differences were observed between the chalazion and nonchalazion groups in tear meniscus height, BUT, conjunctival redness index, and lipid layer thickness (*p* < 0.05). The MG loss subgroup indicated statistically significant differences from the non‐MG loss subgroup in chalazion recurrence frequency, surgical history, tear meniscus height, BUT, conjunctival redness index, and lipid layer thickness (*p* < 0.05). All differential indicators demonstrated no collinearity (VIF ≤ 10, tolerance ≥ 0.1). Logistic regression identified chalazion recurrence frequency and surgical history as independent risk factors for meibomian gland loss. Pearson’s analysis revealed negative correlations between meibomian gland loss severity and both Schirmer’s test results (*r*1 = −0.761) and BUT (*r*2 = −0.543), and a positive correlation with FL scores (*r*3 = 0.752) (all *p* < 0.05).

**Conclusion:**

Retrospective analysis revealed that a proportion of patients with meibomian gland loss had prior chalazion history, demonstrating distinct ocular surface characteristics compared to nonchalazion patients. Recurrent chalazion episodes and surgical history were identified as significant contributors to meibomian gland loss, with glandular loss severity showing strong correlations with DED clinical parameters. These findings highlight the substantial impact of recurrent chalazion and surgical interventions on meibomian gland dysfunction and DED development, emphasizing the need for early clinical recognition and proactive management.

## 1. Introduction

Chalazion, a common ophthalmic condition, is a chronic inflammatory granuloma caused by obstruction of the meibomian gland duct, leading to secretory retention. Children and young adults with active metabolism and vigorous glandular secretion represent high‐risk populations [1–3]. Current clinical treatments include conservative management (antibiotic ointments/eye drops, warm compresses), corticosteroid injections, intense pulsed light therapy, and incision/curettage. Small chalazia (< 3 mm) typically receive conservative or light therapy, while larger lesions (> 3 mm) undergo surgical excision, both of which demonstrate good efficacy [4, 5]. However, long‐term follow‐up reveals suboptimal recovery in some patients, potentially compromising prognosis and quality of life. Therefore, evaluating and addressing long‐term outcomes is crucial for improving patient well‐being.

Recent studies have demonstrated that patients with chalazion exhibit an elevated risk of developing subsequent meibomian gland loss and dry eye disease (DED) [6]. The meibomian glands, specialized sebaceous glands with tubular structures located in the tarsal plates, secrete lipids that form the ocular surface’s protective barrier against microbial and organic insults [7, 8]. Meibomian gland loss may result from physiological degeneration, pathological factors (e.g., conjunctivitis, Sjögren syndrome, and keratitis), and environmental or behavioral factors (e.g., excessive eye use and dry air) [9]. Besides, therapeutic interventions, including medications and surgical procedures, may also contribute to glandular loss. Evidence suggests that corneal transplantation and postoperative medications can destabilize the tear film and alter meibomian gland structure and function [10]. The glandular lipids constitute the tear film’s lipid layer, reducing evaporation rates. Gland loss decreases lipid secretion, accelerates tear evaporation, and compromises tear film stability, ultimately leading to DED [11].

DED is a prevalent ocular surface disorder with a global prevalence of 5%–34%. Its pathogenesis involves multiple factors related to tears and the ocular surface, manifesting as ocular discomfort, tear film instability, visual disturbances, increased tear osmolarity, and subclinical inflammation [12, 13]. DED can be classified into aqueous‐deficient DED (reduced tear production) and evaporative DED (increased tear evaporation) based on pathological mechanisms [14]. Study indicates that meibomian gland dysfunction (MGD), characterized by terminal duct obstruction and/or abnormal glandular secretions, contributes to evaporative DED by destabilizing the tear film and causing ocular discomfort [15]. Meibomian gland loss represents a key pathological basis for MGD. While substantial evidence confirms the association between MGD and DED development/progression, limited data exist regarding influencing factors of gland loss and its correlation with DED clinical parameters. This study investigates the relationship between chalazion history and meibomian gland loss/DED, while analyzing correlations between gland loss severity and DED indicators, aiming to provide theoretical foundations for early targeted interventions.

## 2. Materials and Methods

### 2.1. Study Subjects

This study was a single‐center retrospective case–control observational study. Patients who visited the ophthalmology outpatient clinic of the hospital from June 2021 to June 2024 were selected. An initial screening identified 182 cases, and 32 cases were excluded (11 cases with other ocular surgical history, 5 pregnant women, 8 cases with severe organ dysfunction, and 8 cases with mental disorders). Finally, 150 cases were enrolled (60 in the previous chalazion group and 90 in the nonchalazion group), all of whom were consecutive patients who visited during the same period. Based on *α* = 0.05, *β* = 0.2, and an intergroup difference of 15%, the calculated required sample size was ≥ 150 cases; the final enrollment of 150 cases in this study met the sample size requirement. This single‐center retrospective observational analysis included 60 patients with chalazion and 90 control patients without chalazion from June 2021 to June 2024. The study protocol was approved by the hospital ethics committee and conducted in accordance with the ethical standards of the 1964 *Declaration of Helsinki* and its subsequent amendments. As a retrospective study with anonymized patient data, informed consent was waived.

### 2.2. Inclusion and Exclusion Criteria

Inclusion criteria include the following: (1) Patients in the chalazion group met diagnostic standards from the *Ocular Surface Disease and Anti-Glaucoma Medications: Various features, Diagnosis, and Management Guidelines* [16], confirmed through clinical and imaging examinations; (2) only one eye per participant was included; (3) age ≥ 18 years; (4) no concurrent ocular diseases; and (5) no psychiatric disorders. Exclusion criteria include the following: (1) malignancies; (2) contraindications to study‐related examinations; (3) severe cardiac/hepatic/renal dysfunction; (4) active systemic infections; (5) other ocular surface diseases or surgical history; (6) severe neurological disorders or cognitive impairment; (7) pregnant or lactating women; (8) patients with a history of topical ocular medication use > 3 months; and (9) patients with long‐term exposure to dry/dusty environments.

### 2.3. General Characteristics

This retrospective study collected all patient data through the medical record system, including gender, age, medical history (diabetes), patients met the diagnostic criteria from the *2019 ESC Guidelines on diabetes, pre-diabetes, and cardiovascular diseases developed in collaboration with the EASD* [17], hypertensive patients met the criteria from *Blood pressure and the new ACC/AHA hypertension guidelines* [18], disease duration (obtained by professional physicians using scientific communication techniques), smoking history (≥ 100 cigarettes in the past year), drinking history (≥ 1 drinking session per week for 5 consecutive months), body mass index (BMI) [BMI = weight(kg)/height^2^(m^2^)], surgical history, type of surgery (incision and curettage/intense pulsed light/glucocorticoid injection), incision location (eyelid margin/transconjunctival), surgeon’s professional title, postoperative warm compress compliance, and duration of topical hormone use.

### 2.4. Comprehensive Ocular Surface Examination

The diagnosis of DED was in accordance with the relevant criteria in the “TFOS DEWS II Diagnostic Methodology Report” [19] and was comprehensively evaluated in combination with the Ocular Surface Disease Index (OSDI) [20]. All participants underwent comprehensive ocular surface examination using a noninvasive ocular surface analyzer (Model: Keratograph 5M, Oculus, Germany). The examination procedure was as follows: Subjects were instructed to position their eyes close to the Placido ring on the holder and focus on the red indicator light at the center of the ring. After blinking 2‐3 times, they maintained eye opening while the physician rapidly captured images using a foot pedal. Both upper and lower eyelids were everted for meibomian gland imaging. The measured parameters included the following:

1. Tear Meniscus Height (TMH): Measurements were taken at the 6 o’clock position with a critical value of 0.2 mm. Three measurements were averaged for the final result, with simultaneous observation of tear meniscus continuity.
2. Tear Break‐Up Time (BUT): Fluorescent test strips were used after subjects blinked 3‐4 times. Under cobalt blue light from a slit lamp, the duration from the last blink to the first tear film break‐up point was recorded. The test was repeated three times and averaged.
3. Lipid Layer Observation: The instrument was focused on the tear film lipid layer, and a 5–8‐s video was recorded during natural blinking to assess lipid layer thickness (LLT).
4. Conjunctival Redness Analysis: The instrument was adjusted to obtain the conjunctival redness index.
5. Meibomian Gland Analysis: Infrared light was used to observe the morphology of the meibomian glands. After photographing, the system automatically analyzed the images and generated quantitative values and scores. The Keratograph 5M infrared meibomian gland standardized scoring system (0–3 points) was adopted [21]: 0 points indicated no loss of meibomian glands; 1 point indicated loss of meibomian glands ≤ 1/3; 2 points indicated 1/3 < loss of meibomian glands ≤ 2/3; and 3 points indicated loss of meibomian glands > 2/3.
6. Meibomian Gland Orifices: Evaluated as unobstructed or obstructed.
7. Fluorescein Staining (FL) Test: Sodium fluorescein strips were used to stain the cornea, with observation under cobalt blue filter slit lamp.

Following the ocular surface examination, all subjects underwent Schirmer’s test (SIT) to measure bilateral tear secretion. The same specialized ophthalmic technician performed all examinations.

### 2.5. Laboratory Testing

Fasting peripheral venous blood samples (4 mL) were collected from all participants using vacuum tubes. Samples were promptly delivered to the laboratory and centrifuged at 1900 rpm for 15 min at room temperature to obtain serum and plasma, which were stored at −80°C until analysis.

Complete blood count testing was performed using a five‐part hematology analyzer (Model: BC5300, Mindray, China) by trained technicians following standardized protocols. Red blood cell count, neutrophil count, and hemoglobin concentration were recorded within 4 h of sample collection.

Blood glucose testing was performed using an open automated biochemistry analyzer (Model: Roche Modular P800, Switzerland) and an automated hemoglobin analyzer (Model: HLC‐723 G7, TOSOH, Japan). Fasting blood glucose (FBG) was measured via the glucose oxidase method, while glycated hemoglobin (HbA1c) was quantified through high‐performance liquid chromatography. All analyses were completed within 4 h of sample collection.

### 2.6. Statistical Analysis

Data were analyzed using IBM SPSS 27.0 (IBM Corp., Armonk, N.Y., USA). Categorical data were presented as [*n*] and analyzed by the chi‐square test. Continuous variables were tested for normality using the Shapiro–Wilk test: Non‐normally distributed data were expressed as median and interquartile range [*M*(*P*25, *P*75)], while normally distributed data were presented as mean ± SD and analyzed by *t*‐test. Statistical significance was set at *p* < 0.05. The Pearson correlation coefficient was used to analyze the relationship between meibomian gland loss and DED, with linear regression modeling the association. GraphPad Prism 8.3 was used for statistical analysis, with a significance level of *α* = 0.05. Collinearity was determined to be absent when the variance inflation factor (VIF) was ≤ 10 and the tolerance was ≥ 0.1.

## 3. Results

### 3.1. General Characteristics and Laboratory Findings

#### 3.1.1. Baseline Characteristics

**FIGURE 1:**
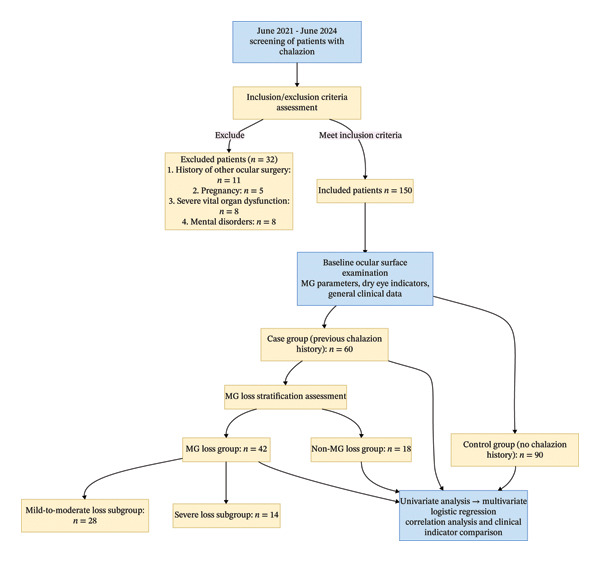
Patient screening and grouping flowchart.

No statistically significant differences were observed between the chalazion and nonchalazion groups regarding gender, age, BMI, smoking history, alcohol consumption, medical history, intraocular pressure, visual acuity, or other ocular parameters (*χ* ^2^1 = 1.094, *p* = 0.296; *Z*1 = −0.732, *p* = 0.464; *Z*2 = −1.643, *p* = 0.100; *χ* ^2^2 = 0.008, *p* = 0.931; *χ* ^2^3 = 0.033, *p* = 0.856; *χ* ^2^4 = 0.040, *p* = 0.841; *χ* ^2^5 = 0.047, *p* = 0.829; *t*1 = 1.838, *p* = 0.068; *t*2 = 1.305, *p* = 0.194; Table 1, Figure 1).

**TABLE 1:** Comparison of baseline characteristics and ocular parameters between chalazion and nonchalazion groups.

Variable		Chalazion group (*n* = 60)	Nonchalazion group (*n* = 90)	*χ* ^2^/*Z*/*t*	*p*
Gender (*n*)	Male	33	51	1.094	0.296
	Female	27	39		

Age (years, *M* [P25, P75])		70.00 (64.00, 73.00)	69.00 (66.80, 74.00)	−0.732	0.464

BMI (kg·m^−2^, *M* [P25, P75])		23.74 (21.60, 25.40)	24.29 (23.00, 25.70)	−1.643	0.100

Smoking history		11	16	0.008	0.931

Alcohol history		10	14	0.033	0.856

Medical history	Hypertension	8	11	0.040	0.841
	Diabetes	6	10	0.047	0.829

Visual acuity		4.70 ± 0.29	4.78 ± 0.24	1.838	0.068

Intraocular pressure (mmHg)		21.05 ± 4.16	20.17 ± 3.97	1.305	0.194

### 3.2. Laboratory and Ocular Surface Examination Results

**FIGURE 2:**
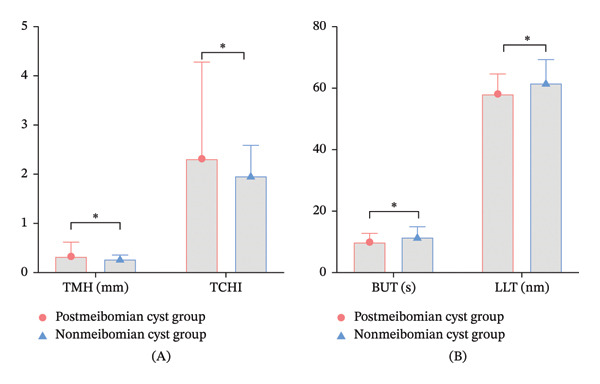
Comparison of ocular surface examination results between the two groups ((A) TMH and TCHI in chalazion vs nonchalazion groups; (B) BUT and LLT in chalazion vs nonchalazion groups; ^∗^ *p* < 0.05). Tear meniscus height (TMH), conjunctival redness index (TCHI), break‐up time (BUT), lipid layer thickness (LLT).

No statistically significant differences were observed between the chalazion and nonchalazion groups in red blood cell count, neutrophil count, hemoglobin concentration, FBG, or HbA1c levels (*t*1 = 0.540, *p* = 0.590; *t*2 = 0.921, *p* = 0.359; *t*3 = 0.416, *p* = 0.678; *t*4 = 1.088, *p* = 0.278; *t*5 = 0.613, *p* = 0.541; Table 2). As shown in Figure 2, the chalazion group demonstrated significantly higher TMH and conjunctival hyperemia index (TCHI) (*t*6 = 3.221, *p* = 0.002; *t*7 = 2.903, *p* = 0.004) compared to the nonchalazion group. Conversely, the chalazion group showed significantly lower tear BUT and LLT (*t*8 = 3.186, *p* = 0.002; *t*9 = 3.014, *p* = 0.003).

**TABLE 2:** Comparison of laboratory findings between chalazion and nonchalazion groups.

Parameter	Chalazion group (*n* = 60)	Nonchalazion group (*n *= 90)	*t*	*p*
RBC count (× 10^12^/L)	3.67 ± 0.87	3.75 ± 0.90	0.540	0.590
Neutrophil count (× 10^9^/L)	6.32 ± 1.67	6.04 ± 1.92	0.921	0.359
Hemoglobin (g/L)	340.92 ± 25.84	342.86 ± 29.35	0.416	0.678
FBG (mmol/L)	4.62 ± 1.31	4.85 ± 1.24	1.088	0.278
HbA1c (%)	5.72 ± 1.03	5.84 ± 1.26	0.613	0.541

### 3.3. Comparison of Clinical Characteristics Between MG Loss and Non‐MG Loss Groups

As shown in Table 3, the proportion of patients with chalazion recurrence frequency of more than 2 times was higher in the MG loss group than in the non‐MG loss group (*χ* ^2^1 = 8.927, *p* = 0.003). The proportion of patients with a history of chalazion surgery of 2 times or more was higher in the MG loss group than in the non‐MG loss group (*χ* ^2^2 = 6.450, *p* = 0.011). The MG loss group exhibited significantly higher TMH (*t*1 = 2.670, *p* = 0.010) and TCHI (*t*2 = 2.743, *p* = 0.008), but lower BUT (*t*3 = 12.229, *p* < 0.001) and LLT (*t*4 = 4.811, *p* < 0.001) compared to the non‐MG loss group. No significant differences were observed between groups regarding age, BMI, type of surgery, incision location, surgeon’s professional title, postoperative warm compress compliance, duration of topical hormone use, smoking history, alcohol consumption, hypertension, diabetes, tear meniscus continuity, or meibomian gland orifice obstruction (*p* > 0.05).

**TABLE 3:** Comparison of clinical characteristics between MG loss and non‐MG loss groups.

Clinical characteristics		MG loss group (*n* = 42)	Non‐MG loss group (*n* = 18)	*χ* ^2^/*t*/*Z*	*p*
Age (years)	> 60	38	16	0.079	0.778
	≤ 60	4	2		

BMI (kg/m^2^)		23.740 (21.8,25.2)	23.705 (21.2,25.5)	−0.105	0.916

Chalazion episodes	1‐2	15	14	8.927	0.003
	> 2	27	4		

Surgical interventions	0/1	23	16	6.450	0.011
	≥ 2	19	2		

Type of surgery	Incision and curettage	24	10	0.056	0.972
	Intense pulsed light	12	5		
	Glucocorticoid injection	6	3		

Incision location	Eyelid margin incision	18	7	0.116	0.944
	Transconjunctival incision	5	2		
	No incision	19	9		

Surgeon’s professional title	Senior title	20	8	0.260	0.878
	Intermediate title	15	6		
	Junior title	7	4		

Postoperative warm compress compliance	Good	32	12	0.199	0.656
	Poor	10	6		

Duration of topical hormone use	< 1 week	15	6	1.012	0.603
	1–2 weeks	20	7		
	> 2 weeks	7	5		
Smoking (*n*)		8	3	0.021	0.884

Alcohol use (*n*)		8	2	0.143	0.706

Hypertension	Yes	6	2	0.007	0.934
	No	36	16		

Diabetes	Yes	5	1	0.079	0.778
	No	37	17		

Tear meniscus continuity (*n*)	Continuous	23	13	1.601	0.206
	Discontinuous	19	5		

Meibomian gland orifices (*n*)	Unobstructed	25	15	3.214	0.073
	Obstructed	17	3		

Tear meniscus height (mm)		0.40 ± 0.13	0.31 ± 0.09	2.670	0.010

BUT(s)		7.04 ± 1.26	12.38 ± 2.09	12.229	< 0.001

Conjunctival hyperemia index		2.59 ± 0.85	1.99 ± 0.56	2.743	0.008

Lipid layer thickness (nm)		54.92 ± 4.77	61.57 ± 5.22	4.811	< 0.001

### 3.4. Logistic Regression Analysis of Factors Influencing Meibomian Gland Loss in Chalazion Patients

As shown in Table 4, logistic regression analysis of differential parameters identified chalazion recurrence frequency and surgical history as significant factors for secondary meibomian gland loss. Specifically, more than 2 chalazion episodes (OR = 6.33, *p* = 0.016) and more than 2 surgical interventions (OR = 9.75, *p* = 0.039) emerged as independent risk factors. TMH (OR = 0.10, *p* = 0.030) and LLT (OR = 0.68, *p* = 0.001) were protective factors against gland loss.

**TABLE 4:** Logistic regression analysis of factors influencing secondary meibomian gland loss in chalazion patients.

Factor	*β*	SE	*Z*	*p*	OR	*OR* 95% CI
Chalazion episodes > 2	1.85	0.77	2.41	0.016	6.33	1.41–28.39
Surgical interventions ≥ 2	2.28	1.11	2.06	0.039	9.75	1.12–85.16
Tear meniscus height	5.49	3.03	1.81	0.070	241.64	0.64–91472.53
BUT	−2.28	1.05	−2.16	0.030	0.10	0.01–0.81
Conjunctival hyperemia index	0.88	0.49	1.80	0.072	2.42	0.92–6.33
Lipid layer thickness	−0.38	0.12	−3.23	0.001	0.68	0.54–0.86

### 3.5. Correlation Between Meibomian Gland Loss Severity and DED Clinical Parameters

#### 3.5.1. Meibomian Gland Loss Severity

Ocular surface analysis revealed that among 42 patients with meibomian gland loss, 11 showed < 1/3 gland loss, 17 demonstrated 1/3–2/3 loss (classified as the mild–moderate group), and 14 exhibited > 2/3 loss (classified as the severe group).

### 3.6. Comparison of DED Parameters Between Severity Groups

**FIGURE 3:**
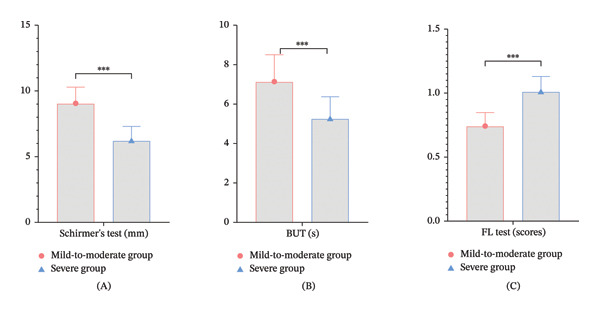
Comparison of DED clinical parameters between the two groups ((A) Schirmer’s test results in mild–moderate vs severe groups; (B) BUT values in mild–moderate vs severe groups; (C) FL test scores in mild–moderate vs severe groups; ^∗∗∗^ *p* < 0.05). Dry eye disease (DED), break‐up time (BUT), ocular surface fluorescein staining (FL).

As shown in Figure 3, the severe group displayed significantly lower Schirmer test results (6.23 ± 1.07 vs 9.04 ± 1.25) and BUT (5.27 ± 1.12 vs 7.13 ± 1.39), along with higher FL scores (1.01 ± 0.12 vs 0.74 ± 0.11) compared to the mild–moderate group (*t*1 = 7.187, *t*2 = 4.343, *t*3 = 7.277, respectively; all *p* < 0.05).

### 3.7. Relationship Between Meibomian Gland Loss Severity and DED Clinical Parameters

**FIGURE 4:**
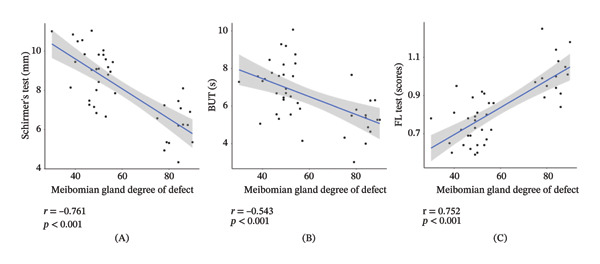
Correlation between meibomian gland loss severity and patients’ Schirmer’s test, BUT, and FL test scores ((A) correlation between gland loss severity and Schirmer’s test results; (B) correlation between gland loss severity and BUT values; (C) correlation between gland loss severity and FL test scores). Break‐up time (BUT), ocular surface fluorescein staining (FL).

Pearson’s correlation analysis revealed significant linear relationships between meibomian gland loss severity and all DED parameters (visualized in Figure 4). Specifically, gland loss showed strong negative correlations with Schirmer’s test (*r* = −0.761) and BUT (*r* = −0.543), while demonstrating a strong positive correlation with FL scores (*r* = 0.752). These findings indicate clinically significant associations between gland loss severity and DED manifestations.

## 4. Discussion

The meibomian glands, as target tissues for sex hormones, are directly influenced by hormonal regulation of lipid secretion, explaining the higher incidence of chalazion in hormonally active populations (typically aged 10–29 years). The widespread use of digital devices has prolonged near‐work duration, and such visual habits contribute to the rising prevalence of ocular surface disease [22, 23]. Recent clinical investigations through medical history tracing have established chalazion as a significant factor in both meibomian gland loss and DED pathogenesis.

Meibomian gland loss or atrophy represents a common clinical manifestation of MGD. Previous studies investigating contributing factors have identified age, ocular medication use, eye surgery, and environmental elements as relevant determinants [24]. Our logistic regression analysis demonstrated that chalazion recurrence frequency constitutes an independent risk factor for secondary gland loss. Recurrent chalazion induces acinar epithelial keratinization and ductal fibrosis through inflammatory pathways mediated by IL‐1β, MMP‐9, IL‐6, TNF‐α, etc., leading to irreversible loss of glands. Relevant data show that meibomian gland acinar density in patients with recurrent episodes is reduced by more than 50% compared with healthy populations [25]. Meanwhile, incision and curettage of chalazion has drawbacks, such as accidental injury to the lacrimal canaliculus, incomplete removal, and postoperative entropion, with a relatively high risk of postoperative recurrence [26]. This study further found that multiple surgeries are also an important influencing factor for meibomian gland loss. Repeated surgical trauma at the same site interrupts the continuity between meibomian gland acini and ducts, accelerating acinar cell apoptosis. In addition, surgical approaches, such as eyelid margin incisions, can easily damage the nerve innervation and microvascular supply around the glands. Poor incision healing with scar hyperplasia can also block local microcirculation, interfere with acinar cell energy metabolism, and induce cell senescence, ultimately substantially increasing the risk of meibomian gland loss. As clinical attention to meibomian gland deficiency has increased, studies have confirmed that such ocular surface damage can further induce DED [27].

DED is an ocular surface disease characterized by tear film instability and ocular surface damage caused by abnormal tear quality/quantity or tear film dynamics disorders [28]. Its pathogenesis is closely linked to tear film instability, ocular surface inflammation, tear hyperosmolarity, and abnormalities in neuropathic pain. Among these, the shortening of tear film BUT due to tear film instability is one of the core mechanisms underlying DED. The tear film is composed of a superficial lipid layer, middle aqueous layer, and inner mucin layer; alterations in any of these components can disrupt tear film stability [29, 30]. The meibomian glands primarily secrete the lipid layer. During blinking, lipids are pushed to the corneal surface to participate in tear film formation, while reducing tear evaporation and maintaining watertight closure of the eyelids, serving as a key structure for ensuring tear film stability [31]. The data in this study showed that the TMH and conjunctival redness index were significantly elevated in the previous chalazion group, while BUT and LLT were significantly decreased, with statistically significant differences between the two groups. The core reason lies in the chronic ocular surface injury and MGD caused by the chalazion history. Insufficient meibum secretion thins the tear film lipid layer, leading to excessive tear evaporation and increased osmotic pressure, thereby shortening BUT. The elevated TMH in this group results from reflex compensatory hypersecretion of the lacrimal gland stimulated by chronic ocular surface inflammation, forming a dynamic imbalance with excessive tear evaporation, which represents a hidden manifestation of early dry eye. Clinical studies have confirmed that abnormal lipid layer quality/quantity is the main pathological basis for evaporative DED, often induced by factors, such as MGD, blepharitis, and visual display terminal syndrome, among which MGD is the core causative factor of DED [32, 33]. In this study, patients were divided into mild‐to‐moderate and severe groups according to the degree of meibomian gland loss. The results showed that as the degree of gland loss worsened, patients’ dry eye clinical indicators became more significantly abnormal. Pearson’s correlation analysis further confirmed that the degree of meibomian gland loss was significantly negatively correlated with basal tear secretion and BUT, and significantly positively correlated with FL score, fully validating that meibomian gland loss is closely related to DED onset, and that the degree of gland loss can serve as an important indicator for evaluating the severity of DED.

This study has limitations: Although subjects were selected according to predefined inclusion/exclusion criteria, the final cohort selection warrants further discussion, as potential selection bias and confounding factors may affect the results. The research lacks longitudinal follow‐up and comprehensive objective analysis (e.g., insufficient elucidation of chalazion’s mechanistic impact on DED pathogenesis). The retrospective case–control design can only reveal associations and cannot establish causal relationships; it is also impossible to exclude the possibility that preexisting MGD was the cause of chalazion. Single‐center data require multicenter external validation to enhance the generalizability of the conclusions. Confounding factors, such as a history of ocular medication use and environmental exposure, were not fully collected. The sample size in the severe meibomian gland loss subgroup was relatively small, and caution is warranted when interpreting the results. There was heterogeneity in details, such as surgical type, incision, and surgeon experience, with unmeasured confounding factors present. Unaccounted variables (e.g., age and visual habits that may influence gland loss severity) and relatively subjective gland loss assessment methods may compromise accuracy. Therefore, in future clinical trials, objective indicators may be incorporated from multiple dimensions, more authoritative assessment tools may be adopted, multicenter studies may be conducted, and prospective longitudinal cohort studies may be carried out to clarify temporal sequences and causal relationships, with active exclusion of various confounding factors, so as to ensure the accuracy of results and thoroughly explore the impact of chalazion history on meibomian gland loss and DED.

## 5. Conclusion

Retrospective analysis revealed that a proportion of patients with meibomian gland loss had prior chalazion history, demonstrating distinct ocular surface characteristics compared to nonchalazion patients. Recurrent chalazion episodes and surgical interventions were identified as significant contributors to gland loss, with loss severity showing strong correlations with DED clinical parameters. These findings underscore the substantial impact of recurrent chalazion and surgical procedures on MGD and DED development, necessitating early clinical recognition and proactive management.

## Funding

The authors have nothing to report.

## Ethics Statement

This study was reviewed and approved by the Ethics Committee of Shanghai Tenth People’s Hospital (Approval Number: 21K247). Written informed consent was obtained from all participants, and all procedures followed the principles of the Declaration of Helsinki.

## Conflicts of Interest

The authors declare no conflicts of interest.

## Data Availability

The data that support the findings of this study are available upon request from the corresponding author. The data are not publicly available due to privacy or ethical restrictions.
